# Sexual Activity in Heart Failure Patients: Information Needs and Association with Health-Related Quality of Life

**DOI:** 10.3390/ijerph16091570

**Published:** 2019-05-05

**Authors:** Anneleen Baert, Sofie Pardaens, Delphine De Smedt, Paolo Emilio Puddu, Maria Costanza Ciancarelli, Amos Dawodu, Johan De Sutter, Dirk De Bacquer, Els Clays

**Affiliations:** 1 Department of Public Health and Primary Care, Ghent University, 9000 Ghent, Belgium; sofie.pardaens@ugent.be (S.P.); Delphine.desmedt@ugent.be (D.D.S.); Dirk.debacquer@ugent.be (D.D.B.); Els.clays@ugent.be (E.C.); 2 Department of Cardiovascular Sciences, Sapienza University of Rome, 00185 Rome, Italy; Paoloemilio.puddu@uniroma1.it (P.E.P.); mcostanza.ciancarelli@gmail.com (M.C.C.); 3 Department of occupational medicine, Sapienza University of Rome, 00185 Rome, Italy; Amos.dawodu@uniroma1.it; 4 Department of Internal Medicine, Ghent University, 9000 Ghent, Belgium; Johan.DeSutter@AZMMSJ.BE

**Keywords:** heart failure, sexual activity, quality of life, need for counselling

## Abstract

(1) *Background*: the main objective of this study was to investigate information needs concerning sexual activity and experienced sexual problems in heart failure (HF) patients and, in addition, to examine the association between these sexual problems and health-related quality of life (HRQoL); (2) *Methods*: in this cross-sectional study, three self-administered questionnaires were distributed to 77 stable ambulatory HF patients to acquire data on HRQoL, sexual problems, and need for counselling; (3) *Results*: More than half (56.7%) of HF patients experienced a marked decrease or total cessation of sexual activity due to their illness. Additionally, more than one-third perceived a marked decrease or total absence of sexual pleasure (42.5%), interest (32.9%), and constant problems or being unable to perform sexual activity (37.3%). Furthermore, 43.1% of patients experienced an important overall need for counselling concerning sexual activity, with information on relationships (69.2%), symptoms (58.5%), and relaxation (49.2%) being the most desired topics. Multiple linear regression analysis revealed that sexual problems were independently associated with HRQoL, with more sexual problems (*t* = 3.19, *p* < 0.01) being related to poor HRQoL; (4) *Conclusion*: by investigating the experienced problems and counselling needs of HF patients, an alignment between current practice and HF patients’ expectations and needs might be obtained.

## 1. Introduction

One of the most common cardiovascular diseases is heart failure (HF), with 26 million adults worldwide living with this condition [1]. The prevalence of HF depends on the applied definition but is approximately 1–2% of the adult population in developed countries, with prevalence rising to ≥10% among people >70 years of age [2,3]. HF can be defined as an abnormality of the cardiac structure or function, leading to failure of the heart to deliver oxygen at a rate proportionate to the requirements of the metabolizing tissues [4]. As a result, the body will retain sodium and water in order to compensate for this loss, causing symptoms such as breathlessness, orthopnoea, paroxysmal nocturnal dyspnoea, reduced exercise tolerance and fatigue [5,6,7]. 

Improved treatment of HF has resulted in decreased mortality and hospitalization rates [8], and thus, in addition to prolonging survival, increasing self-perceived health-related quality of life (HRQoL) has become a major goal of HF treatment [9]. Although there is no consensus on the precise definition, HRQoL captures a person’s self-perceived impact of a medical condition, its symptoms, and its treatment [10]. It is highly individual and can mean different things to each patient, depending on demographic, psychological, socioeconomic, and other characteristics, in addition to interpretation based on the patient’s own expectations, hopes, and ambitions [11]. Therefore, not only the patients’ perceived level of satisfaction with their physical functioning but also their perceived emotional and social functioning should be taken into account [12], with sexual activity being an important but largely ignored aspect of HRQoL [4].

Notwithstanding that sexual activity is not a main topic of concern for all HF patients, satisfaction with one’s sexual activity is considered important by most HF patients, including those patients at advanced age [13,14]. In a study of 100 HF patients, 52% of men and 38% of women reported that sex was important and that sexual problems reduced their HRQoL [15], psychological well-being, and relationship satisfaction [16,17]. Strikingly, Schwarz et al. (2008) found in a sample of 100 HF patients, that 75% of men and 60% of women were never asked about sexual intimacy, indicating that treatment options are rarely discussed or initiated in clinical practice [14].

The ability of HF patients to participate in sexual activity depends on New York Heart Association (NYHA) functional classification of their affliction, being stabilized, and receiving optimal medical management [18]. Beta Blockers, diuretics, and cardiac glycosides, frequently used in the treatment of HF, are associated with sexual dysfunction [19], as well as common comorbidities such as diabetes, chronic obstructive pulmonary disease (COPD), hypertension, obesity, and depression [20,21]. HF specific factors that are related to sexual problems are HF symptoms such as dyspnoea, fatigue, and activity intolerance [19]. Furthermore, emotional and psychological concerns in HF patients may result in anxiety and fear regarding sexual activity [22,23]. When engaging in sexual activity, symptoms including chest pain, palpitations, shortness of breath, and fatigue are frequently reported [24]. This creates a need for patients to receive counselling concerning sexual functioning and a safe return to being sexually active [25,26,27].

Although patients with HF often consider sexual activity as an essential aspect of HRQoL and studies show that better sexual function is related to a higher overall well-being [13,28], studies concerning this topic are rather limited and are often inconsistent. Therefore, the objectives of this study were to investigate (i) the information need concerning sexual activity and (ii) the experienced sexual problems in HF patients, in addition to examining (iii) factors related to these outcomes and (iv) the relation between sexual problems and HRQoL.

## 2. Materials and Methods 

### 2.1. Data Collection and Study Population 

The current study is a substudy of the European H2020 HeartMan project. Detailed information on this project can be found elsewhere [29]. Patients meeting the inclusion criteria were ischemic and non-ischemic stable HF patients with at least one hospitalization due to their HF (with no hospitalization in the last month), left ventricular ejection fraction (LVEF) <40%, NYHA I–III, good cognitive functioning, and sufficient knowledge of the Dutch or Italian language. Having reduced sexual activity was not a formal criterion for enrolment in the study, since patients with no formal complaints can still benefit from sexual counselling. Patients were recruited between March and December 2018 in three Flemish hospitals (AZ Maria Middelares, OLV Aalst, and UZGent) and one Italian hospital (Rieti General Hospital) who participated in the HeartMan study. The study complied with the principles outlined in the Declaration of Helsinki and was approved by Medical ethics committees of the University Hospital Ghent (Belgium) and the Lazio 1 of San Camillo-Forlanini Hospital in Rome (Italy) as the central ethical committees. All participants signed an informed consent form before completing the questionnaire.

### 2.2. Questionnaires

In order to acquire data on HRQoL, sexual problems, and need for counselling, three self-administered questionnaires were included, after obtaining the necessary permissions and licenses, which are further described in the following paragraphs. In addition, demographic and clinical data, including age, comorbidities, date of HF onset and prescribed medication, were collected from the medical records by a study nurse.

To assess HRQoL, the 21-item disease-specific Minnesota Living with Heart Failure Questionnaire (MLHFQ) was used. This questionnaire covers symptoms and signs relevant to HF such as physical activity, social interaction, work, and emotions [28] and is scored by summation of responses to all 21 items, with a higher score indicating a more impaired HRQoL. There are no official cut-off values, however, based on literature, the following categorization is proposed: <24 (good), 24–45 (average), and >45 (poor) [30]. 

Data on experienced sexual problems were collected using the Sexual Adjustment Scale (SAS). This scale is one of the seven subscales of the Psychosocial Adjustment to Illness Scale (PAIS), designed to evaluate the shift in quality of sexual relations due to current illness or treatment [12]. The SAS consists of six items addressing relationship (intimacy and discussions) and sexuality (sexual interest, frequency of sexual activity, pleasure and satisfaction, and limitations) [31], scored on a four-point Likert scale ranging from 0 (no disturbance) to 3 (marked disturbance). The total score varies from 0 to 18, with a higher score indicating more disturbance experienced [32]. 

Furthermore, the Needs for Counselling scale in HF (NSCS-CHF) was administered to investigate the need for information regarding sexual activity and the preferred methods for delivery of this information [32]. The NSCS-HF consists of two domains. The first domain concerning content, includes five sub-domains: ‘symptoms’ (four items), ‘medication and information’ (four items), ‘relaxation’ (six items), ‘relationship’ (two items), and ‘psychological factors’ (two items). The questions in each domain were answered with a four-point Likert scale ranging from 1 (totally unimportant) to 4 (very important). An average score was calculated ranging from 1 to 4, with an average of 2.5 or more indicating an important need for counseling [32]. The second domain concerning conditions of delivery had three multiple-choice questions with respect to mode of delivery, and a frequency ranking was determined [32]. 

### 2.3. Data Analysis

Data were analyzed using IBM SPSS 25 (IBM Corp., Armonk, NY, USA)). The sumscores for the MLHF, SAS, and the average score for the NSCS-CHF were calculated in accordance with the guidelines of the questionnaires. Descriptive statistics were used to provide an overview of the current situation concerning experienced sexual problems and information needs in HF patients. Prior to each analysis, the distribution of the variables was checked in order to choose correct statistical tests and to identify outliers. Independent samples *t*-tests and ANOVA were used to compare differences regarding clinical characteristics, sexual activity, and HRQoL, since the assumptions of normality for this test were met. Additionally, Pearson correlation was calculated between the three patient-reported outcomes (SAS, NSCS-CHF, and MLHFQ), and multiple linear regression analyses were performed with MLHFQ as outcome variable and SAS, age, NYHA functional class, and gender as independent variables.

## 3. Results

### 3.1. Sample Characteristics

A total of 77 patients participated in this study. The study population consisted predominantly of Belgian (75.7%), male (73%) patients with NYHA class II (72.3%), and a mean age of 62.3 (SD = 10.6). There was an almost perfect balance between ischemic (50.7%) and non-ischemic (49.3%) HF. A vast majority of patients were prescribed beta blockers (93.2%) (of which, 71.6% of generation 2, and 28.4% of generation 3) in combination with aldosterone antagonists (65.8%) and angiotensin-converting-enzyme (ACE) inhibitors or angiotensin receptor blockers (ARBs) (65.8%). In addition, more than three out of four (76.7%) patients made use of diuretics, whereas only 8.2% used ivabradine. Furthermore, the majority (78.6%) of the patients was at least overweight, with 32.8% suffering from obesity. The most common comorbidity and risk factors were hyperlipidemia (71.6%), followed by hypertension (55.4%) and diabetes (39.7%). Table 1 provides a detailed overview of the demographic and clinical characteristics of the respondents.

### 3.2. Sexual Problems and Need for Counseling 

**Figure 1:**
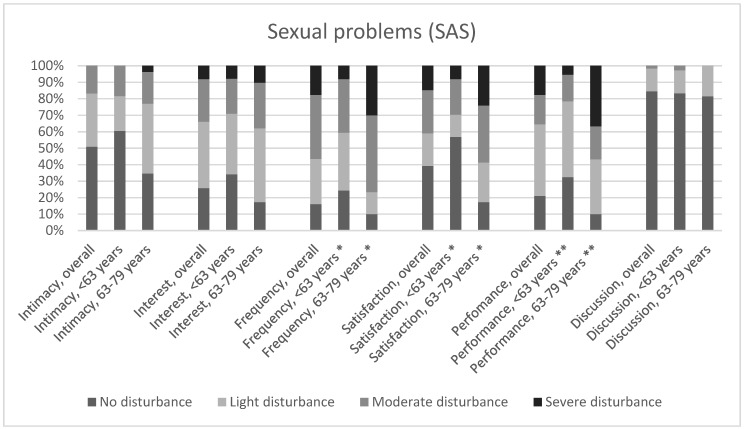
Experienced sexual problems. * Significant difference based on age at *p* < 0.01 level; ** significant difference based on age at *p* < 0.001 level. SAS, sexual adjustment scale.

A mean total score of 5.9 (SD = 3.9) out of 18 was found for experienced sexual problems, and the higher the score, the more intense the disturbance experienced. Comparison between groups based on gender, age, and comorbidities, revealed a significant difference, with men, patients younger than 63, and those suffering from hyperlipidemia experiencing more sexual problems (Table 2). Half (50.1%) of the participants indicated at least a light to moderate decrease in intimacy with their partner due to HF, whereas the others experienced no change (Figure 1). Furthermore, patients reported a moderate to severe decrease in interest in (32.9%), frequency of (56.7%), and satisfaction of (42.5%) sexual activity since onset of their HF. In addition, 40.3% of patients reported some limitations in sexual activity due to HF, whereas 17.9% experienced constant problems, and 19.4% were unable to perform sexual activity. However, the vast majority (82.5%) indicated to never have arguments with their partners concerning this topic. A significant difference in experienced sexual problems based on age was found between patients younger and those older than 63, with the older ones experiencing more performance problems (*p* < 0.001) and a larger decrease in the frequency (*p* < 0.01) and satisfaction (*p* < 0.01) of sexual activity (Figure 1).

**Figure 2:**
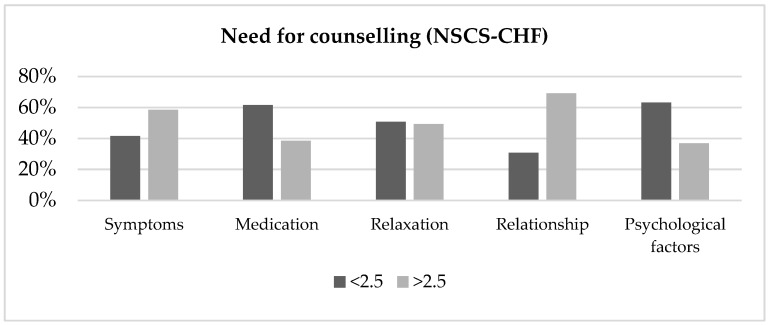
Experienced need for sexual counselling.

More than half (69.2%) of the patients reported an important need for information on how to communicate on feelings, concerns, and possibilities within their relationship, whereas 58.5% were interested in information on how to cope with symptoms associated with HF during sexual activity (Figure 2). 

Furthermore, 49.2% indicated a desire for additional education on the importance of relaxation before and during sexual activity and the influence of variety of factors. In addition, more than one out of three patients felt insufficiently informed about the influence of medication (38.5%) and the influence of anxiety and depression (36.9%) on sexual functioning. Results revealed that 43.1% of participants experienced an important overall need for information (>2.5) concerning sexual activity. Comparison between men and women and the presence or absence of hyperlipidemia indicated a greater information need in male patients and those suffering from this comorbidity (Table 2).

More than half (58.1%) of the patients indicated a conversation with a healthcare provider as the preferred method of information delivery, followed by written information (25.8%) and information accessible through computer (8.1%). Counselling provided through telephone (3.2%) or video/DVD (3.2%) was the least popular. Furthermore, patients favored being counselled by a cardiologist (43.9%) or general practitioner (24.6%), over sexologists (15.8%), nurses (10.6%), or psychologists (5.3%). Additionally, nearly half of the patients (47.5%) preferred to receive additional information on this topic in the presence of their partner, whereas 44.1% preferred it without their spouse. Only 6.8% were interested to discuss this topic in the presence of fellow HF patients. 

### 3.3. Health-Related Quality of Life

Results indicated a mean score of 31.6 (SD = 20.5) for the MLHFQ, with 43.2% experiencing a good (<24), 28.4% an average (score 24–45), and 28.4% a poor (score >45) HRQoL. A significant difference between groups for HRQoL could be identified on the basis of NYHA functional class, with patients in NYHA III experiencing the worst HRQoL, followed by patients in NYHA II and NYHA I. Furthermore, a borderline significant difference was found between men and women, with men showing a worse HRQoL. 

On the basis of Pearson correlation analyses, a significant correlation was found between sexual problems, measured by SAS, and HRQoL (*r =* 0.39, *p* < 0.01), in addition to a significant correlation found between sexual problems and the need for counselling in patients (*r =* 0.27, *p* < 0.05). The results indicated that an increase in sexual problems was associated with a worse HRQoL and an increase in information need. In contrast, no correlation was found between the need for counselling and HRQoL (Table 3).

Furthermore, multiple linear regression analysis (Table 4) revealed that the SAS score was independently associated with the MLHFQ score, with more sexual problems (*t* = 3.19, *p* < 0.01) increasing the chance of a worse HRQoL. Additionally, being male was associated with a worse HRQoL (*t* = 2.24, *p =* 0.03). However, no significant independent association was found for NYHA functional class or age.

## 4. Discussion

The main objective of this study was to investigate the information needs concerning sexual activity and the experienced sexual problems in HF patients. In addition, the relation between these sexual problems and HRQoL was examined. The results from our study indicate that more than one out of three patients experience an important need for sexual counselling, and a variety of sexual problems were reported, with these problems being significantly associated with a decrease in HRQoL. 

The results from our study indicate that symptoms of HF may affect the sexual relations of HF patients, with 40% perceiving a marked decrease or total absence of sexual pleasure, 35.9% indicating a decrease or disappearance of interest, and 35% experiencing constant problems or being unable to perform sexual activity. Furthermore, 56.6% of patients experienced a marked decrease in frequency or total cessation of sexual activity. These findings are in line with findings from Van Driel et al. (2014) [30] and Jaarsma (2002) [13]. Differences between studies might be due to a difference in distribution between the NYHA functional classes and a lower mean LVEF in the study of Van Driel et al. (2014), and a higher mean age and NYHA functional class in the study performed by Jaarsma (2002). Furthermore, the aspect least impacted by HF appeared to be the relation with the partner, since the majority of our participants indicated to have no or only a slight loss in intimacy (83%) and no discussion concerning the topic of sexual activity with their partner (84.5%). This finding indicates that frequency of sexual contact and satisfaction with sexual activity, does not necessarily have a negative impact on other aspects of the relationship. 

Evaluation of HF patients’ needs indicated patients require information concerning relationship (how to communicate on feelings, concerns, and possibilities), symptoms, relaxation, medication, and psychological factors and its importance. Furthermore, more than half of the patients indicated a conversation with a healthcare provider as the preferred method of information delivery, followed by written information. These results are in line with the findings of Van Driel et al. (2014); however, an important difference was found regarding the favored healthcare provider for delivery of this information. Results by Van Driel et al. (2014) indicate a preference for HF nurses, followed by general practitioners and cardiologists, whereas our study population showed a clear preference for counselling provided by a cardiologist (41.8%) or a general practitioner (23.6%), with only 10.9% favoring HF nurses. These results confirm the statement by Steinke and Jaarsma (2015) that the responsibility for providing sexual counselling is shared by physicians, nurses, physical therapists, rehabilitation staff, and others [18]. 

A difference in sexual problems, need for information on sexual activity, and HRQoL was found in relation to gender, with men experiencing more problems, desiring overall more information, and having a worse HRQoL than women. This might be the result of the greater importance men attribute to sex and their tendency to report problems of sexual dysfunction more commonly, when compared to women [15,33]. However, the existing literature is inconclusive on the differences in HRQoL between genders, since some studies found no association between gender and HRQoL, whereas others found being female or male as a predictor for a reduced HRQoL [34]. Furthermore, patients with hyperlipidemia experienced more sexual problems and an increased information need than those without, which might be explained by the connection between hyperlipidemia and sexual dysfunction in men and women [35,36]. Moreover, a difference in sexual problems and HRQoL was found on the basis of NYHA classification, with a higher NYHA functional class being associated with more sexual problems and a decrease in HRQoL. Since NYHA classification is a way of categorizing the extent of HF and its severity of symptoms [37], with a higher functional class representing more severe symptoms, it is not surprising that this finding is in line with the existing literature [34]. Additionally, our study found a difference in sexual problems based on age. Throughout life, there are age-related changes in sexuality caused by normal hormonal changes, vascular damage, or muscular weakness [38], therefore, it should be acknowledged that not all sexual problems experienced by HF patients are caused by their disease but might be part of the natural aging process [39]. In contrast, no difference in HRQoL based on age was observed, despite previous studies indicating that age was systematically related to HRQoL, with older participants being more satisfied with their lives than their younger counterparts [34,40]. 

Our results indicate that an increase in sexual problems was correlated with a poorer HRQoL and an increase in information needs. This relation was also found in a study by Jaarsma (2002) in 73 HF patients using the ‘ladder of life’ to examine the overall quality of life [13]. Moreover, an independent association was found between gender and sexual problems on one hand and HRQoL on the other, with being male and experiencing more sexual problems being associated with a lower HRQoL. Since the MLHFQ, used to assess HRQoL, also consists of an item concerning the influence of HF on sexual activity, additional analyses were performed to investigate the mediating effect of this item. Nevertheless, the correlation and independent association between sexual problems and HRQoL persisted after omitting this item from the sum score of the MLHFQ (results not shown). However, determining the cause and effect between sexual problems and HRQoL based on these results is not possible, since an increase in sexual problems can have an impact on experienced HRQoL, whereas a better HRQoL may also positively influence sexual relations [13].

### Limitations

The results from this study should be interpreted with some caution, since the questionnaires were administered in the presence of a healthcare provider, which might have resulted in a social desirability bias. However, to limit the response bias, the present healthcare provider clearly stated that all results were confidential and processed anonymously. Furthermore, we should be aware of a selection bias and cultural differences due to the use of a convenience sample in two European countries (Belgium and Italy); nonetheless, statistical analyses showed no differences based on nationality, increasing the generalizability of our results. In addition, no response rate could be determined due to the absence of a screening list, and this study is slightly underpowered for performing multiple regression analyses with four independent variables, since a minimum required sample of 84 was desirable for a medium anticipated effect (0.15), when using a 95% confidence level. 

This study made use of disease-specific measures to evaluate HRQoL and need for sexual counselling, which have generally been shown to be more sensitive to changes than their general counterparts, as they better capture issues which are relevant to these patients; however, they do not allow comparisons with the general population, making interpretation rather difficult [39]. Additionally, due to the homogeneity of the sample, it was not possible to assess the influence of reduced versus preserved LVEF. Moreover, medication prescribed to treat HF is frequently perceived to cause problems with libido or sexual performance [41]. However, the influence of the prescribed medication could not be determined because of the small sample size and the homogeneity in prescribed medication classes in accordance with the European Society of Cardiology (ESC) guidelines [3]. Nevertheless, our study found no influence of beta blocker generation on sexual problems, whereas recent studies indicated that third-generation beta blockers (which show additional vasodilating effects over the second generation) currently used in HF treatment may have less effect on sexual function [14,40]. This discrepancy might be explained by the use of sexual problems (which included experienced limitations) as outcome in this study versus the narrower sexual dysfunctions in other research.

## 5. Conclusions

Almost half of HF patients attributed a marked decrease in frequency or total cessation of sexual activity to their illness, whereas more than one-third perceived a marked decrease or total absence of sexual pleasure, a decrease or disappearance of interest, and constant problems or being unable to perform sexual activity. Furthermore, one out of three patients experienced an important need for counselling concerning sexual activity, with information on relationship, symptoms, and relaxation being the most desired topics. Experiencing sexual problems was significantly associated with the need for counselling and a reduced HRQoL. Further studies are required to explore the current practice of sexual counselling and to reveal how the information can be provided by healthcare providers in agreement with HF patients’ expectations and needs.

## Figures and Tables

**Table 1:** Sample size characteristics.

Characteristics			Valid % (N) or Mean (SD)
Gender (*N* = 74)		Male	73% (54/74)
		Female	27% (20/74)
Age, years (*N* = 74)			62.3 (10.6)
Country (*N* = 74)		Belgium	75.7% (56/74)
		Italy	24.3% (18/74)
NYHA (*N* = 65)		Class I	13.8% (9/67)
		Class II	72.3% (47/67)
		Class III	12.3% (8/67)
BMI ^a^, kg/m² (*N* = 67)		18.5–25	19.4% (13/67)
		25–30	45.8% (32/67)
		30–35	16.4% (11/67)
		>35	16.4% (11/67)
Etiology heart failure (*N* = 69)		Ischemic	50.7% (35/69)
		Non-ischemic	49.3% (34/69)
LVEF ^b^ (*N* = 71)			33.3 (8.1)
Medication (*N* = 73)			
	Beta blocker	Yes	93.2% (68/73)
	Aldosterone antagonist	Yes	65.8% (48/73)
	ACE inhibitor	Yes	56.2% (41/73)
	ARB ^c^	Yes	9.6% (7/73)
	Diuretics	Yes	76.7% (56/73)
	Ivabradine	Yes	8.2% (6/73)
Comorbidities and risk factors			
	Hypertension ^d^ (*N* = 65)	Yes	55.4% (36/65))
	Hyperlipidemia ^e^ (*N* = 67)	Yes	71.6% (48/67)
	Diabetes (*N* = 68)	Yes	39.7% (27/68)
Patient reported outcomes			
	SAS ^f^ (*N* = 69)		5.9 (3.9)
	NSCS-CHF ^g^ (*N* = 65)		2.3 (0.7)
	MLHFQ ^h^ (*N* = 74)		31.6 (20.5)

Continuous variables are presented as mean (SD), while categorical variables are represented by valid% (n). ^a^ body mass index, ^b^ left ventricular ejection fraction, ^c^ angiotensin receptor blockers, ^d^ Hypertension (>140/90 mmHg) or medication, ^e^ Hyperlipidemia (LDL > 115 mg/dL or medication), ^f^ Sexual Adjustment Scale, ^g^ Needs for Sexual Counselling Scale in Heart Failure, ^h^ Minnesota Living with Heart Failure Questionnaire, NYHA, New York Heart Association, ACE, angiotensin-converting-enzyme.

**Table 2:** Comparison between groups for the patient-reported outcomes.

Independent Variable		Sexual Problems (SAS)		Need for Counselling (NSCS-CHF)		Health-Related Quality of Life (MLHFQ)	
		Mean (SD)	*p*-Value	Mean (SD)	*p*-Value	Mean (SD)	*p*-Value
Sex			<0.01 *		0.01 *		0.12
	*Male*	6.72 (3.86)		2.39 (0.68)		29.39 (20.57)	
	*Female*	3.79 (3.12)		1.89 (0.74)		37.7 (19.87)	
Age			<0.01 *		0.74		0.48
	*<63*	4.71 (3.84)		2.23 (0.74)		30.08 (22.56)	
	*63–79*	7.49 (3.44)		2.29 (0.72)		33.47 (17.96)	
Country			0.54		0.15		0.38
	*Belgium*	5.76 (3.85)		2.2 (0.72)		32.82 (21.55)	
	*Italy*	6.47 (4.09)		2.56 (0.74)		27.94 (16.88)	
Beta Blocker generation			0.79		0.87		0.19
	*Generation 2* *Generation 3*	5.86 (3.85) 5.58 (3.5)		2.25 (0.72) 2.29 (0.65)		32.04 (19.9) 24.74 (20.67)	
Etiology HF			0.15		0.25		0.91
	*Ischemic*	6.59 (3.98)		2.12 (0.82)		31.43 (22.64)	
	*Non-ischemic*	5.18 (3.81)		2.34 (0.65)		31.97 (18.03)	
Hypertension			0.48		0.62		0.28
	*Yes*	6.27 (4.03)		2.16 (0.8)		29.11 (21.27)	
	*No*	5.53 (4.03)		2.27 (0.68)		34.72 (19.78)	
Hyperlipidemia			<0.01 *		<0.01 *		0.17
	*Yes*	6.71 (3.91)		2.37 (0.69)		33.04 (21.67)	
	*No*	3.67 (3.31)		1.81 (0.68)		25.58 (13.17)	
Diabetes			0.11		0.48		0.15
	*Yes*	6.88 (3.97)		2.3 (0.77)		36.04 (23.44)	
	*No*	5.26 (3.87)		2.17 (0.72)		28.71 (18.01)	
NYHA			<0.01 *		0.09		<0.01 *
	*Class I* *Class II* *Class III*	2.36 (1.6) 5.73 (3.81) 9.13 (2.8)		1.73 (0.72) 2.29 (0.7) 2.65 (0.88)		20.78 (19.27) 29.55 (18.31) 55.5 (17.19)	

* significant at *p* < 0.01 level. HF, heart failure.

**Table 3:** Pearson correlation.

	NSCS-CHF	MLHFQ
**NSCS-CHF**		0.17
**SAS**	0.27 *	0.39 **

* Significant at *p* < 0.05. ** Significant at *p* < 0.01.

**Table 4:** Multiple linear regression with health-related quality of life (HRQoL) as a dependent outcome variable.

Independent Variable	Health-Related Quality of Life (MLHFQ)			
	Standardized B	*t*	95% CI	Adjusted *p*-Value
Sex	0.31	2.37	2.11–25.19	0.02
Age	−0.01	−0.06	−10.78–10.11	0.95
NYHA	0.15	1.18	−1.98–7.7	0.24
Sexual problems (SAS)	0.42	3.19	0.82–3.58	<0.01

Adjusted R-square of 14.6%.
